# Lower maximum forces on oral structures when using gum-elastic bougie than when using endotracheal tube and stylet during both direct and indirect laryngoscopy by novices: a crossover study using a high-fidelity simulator

**DOI:** 10.1186/s12873-020-00328-9

**Published:** 2020-05-06

**Authors:** Yuko Ono, Kazuaki Shinohara, Jiro Shimada, Shigeaki Inoue, Joji Kotani

**Affiliations:** 1 Department of Disaster and Emergency Medicine, Graduate School of Medicine, Kobe University, 7-5-2 Kusunoki-cho, Chuo-ward, Kobe, 650-0017 Japan; 2 Emergency and Critical Care Medical Center, Fukushima Medical University, 1 Hikarigaoka, Fukushima, 960-1295 Japan; 3 Department of Anesthesiology, Ohta General Hospital Foundation, Ohta Nishinouchi Hospital, 2-5-20 Nishinouchi, Koriyama, 963-8558 Japan

**Keywords:** Airway-related adverse events, Medical student, Teeth injury, Tracheal tube introducer, Video laryngoscope

## Abstract

**Background:**

Applying excessive force during endotracheal intubation (ETI) is associated with several complications, including dental trauma and hemodynamic alterations. A gum-elastic bougie (GEB), a type of tracheal tube introducer, is a useful airway adjunct for patients with poor laryngoscopic views. However, how the use of a GEB affects the force applied during laryngoscopy is unclear. We compared the force applied on the oral structures during ETI performed by novices using the GEB versus an endotracheal tube + stylet.

**Methods:**

This prospective crossover study was conducted from April 2017 to March 2019 in a public medical university in Japan. In total, 209 medical students (4th and 5th grade, mean age of 23.7 ± 2.0 years) without clinical ETI experience were recruited. The participants used either a Macintosh direct laryngoscope (DL) or C-MAC video laryngoscope (VL) in combination with a GEB or stylet to perform ETI on a high-fidelity airway management simulator. The order of the first ETI method was randomized to minimize the learning curve effect. The outcomes of interest were the maximum forces applied on the maxillary incisors and tongue during laryngoscopy. The implanted sensors in the simulator quantified these forces automatically.

**Results:**

The maximum force applied on the maxillary incisors was significantly lower when using a GEB than when using an endotracheal tube + stylet both with the Macintosh DL (39.0 ± 23.3 vs. 47.4 ± 32.6 N, *P* < 0.001) and C-MAC VL (38.9 ± 18.6 vs. 42.0 ± 22.1 N, P < 0.001). Similarly, the force applied on the tongue was significantly lower when using a GEB than when using an endotracheal tube + stylet both with the Macintosh DL (31.9 ± 20.8 vs. 37.8 ± 22.2 N, P < 0.001) and C-MAC VL (35.2 ± 17.5 vs. 38.4 ± 17.5 N, P < 0.001).

**Conclusions:**

Compared with the use of an endotracheal tube + stylet, the use of a GEB was associated with lower maximum forces on the oral structures during both direct and indirect laryngoscopy performed by novices. Our results suggest the expanded role of a GEB beyond an airway adjunct for difficult airways.

## Background

Effective, timely airway management can be the key to saving the lives of critically ill patients. Endotracheal intubation (ETI) is the gold standard definitive airway management technique and one of the most frequently performed procedures in the emergency department (ED). Therefore, ETI is a vital skill for emergency physicians. However, ETI can also cause severe complications including esophageal intubation, hypotension, hypoxemia, bradycardia, dysrhythmia, cardiac arrest, and dental trauma, especially when performed in ED settings [1–5]. Of these ETI-related adverse events, hemodynamic alterations such as cardiac arrest, bradycardia, and dysrhythmia as well as dental injury are associated with excess force applied on the oral structures during laryngoscopy [6–8]. Therefore, glottic exposure with a lower force is of paramount importance for conducting safer ETI in the ED.

The ED is also an important place for teaching airway management skills to novice physicians. For example, approximately one-third to one-half of ETI procedures are currently performed by junior residents in the EDs in developed countries [1, 9–11]. Therefore, initiating novices into safer ETI is necessary to ensure patient safety and improve ETI outcomes. For this purpose, senior physicians must employ effective laryngoscopes and airway adjuncts when supervising ETI performed by novices.

Glottic exposure is likely to be insufficient if laryngoscopy is performed by inexperienced clinicians. Accordingly, inexperienced laryngoscopists tend to apply more force to the incisors and tongue than do experienced laryngoscopists [8]. A gum-elastic bougie (GEB), which is a type of tracheal tube introducer, is a useful airway adjunct for patients with poor laryngeal views [12, 13] or can be used as a rescue device when initial intubation attempts fail [14, 15]. In previous studies, the use of a GEB reduced the force of laryngoscopy in a simulated difficult airway with limited glottic visualization among anesthesia providers [16]. Therefore, we postulated that the use of a GEB would improve the force applied on the oral structures during laryngoscopy performed by novices. Because no previous study has adequately addressed this clinical question, and because direct clinical evaluations are difficult and unethical, we tested our hypothesis by conducting a crossover study using a high-fidelity simulator.

## Methods

### Study design, setting, and participants

This prospective crossover study using a high-fidelity simulator was conducted from April 2017 to March 2019 at a simulation laboratory in a public medical university in Japan. The crossover design enabled each study participant to serve as his or her own control, thereby removing the effect of both measured and unmeasured confounders [17]. After approval by the Institutional Review Board at Fukushima Medical University (no 2990), 4th- and 5th-grade medical students who had not been trained to use either a direct or indirect laryngoscope were recruited in this study. Past studies also employed medical students as a model of novice airway management [18, 19]. Written informed consent was obtained from each participant, and their characteristics such as age and sex were documented.

### Devices

All ETI attempts were performed using the following devices: Macintosh direct laryngoscope (DL) with No. 3 blade (Cat. Nos. 3000.500.10 and 2971.150.20; Smiths Medical, Minneapolis, MN, USA) and C-MAC video laryngoscope (VL) with No. 3 blade and monitor (Cat. Nos. 8403AX/AXC and 8403ZXK; Karl Storz, Tuttlingen, Germany), Portex® tracheal tubes with an internal diameter of 7.0 mm (Cat. No. 100/199/070; Smiths Medical), and the manufacturer’s stylet (Portex® stylet, Cat. No. 100/120/200; Smiths Medical) and GEB (Portex® Tracheal Tube Introducer 15-Fr, Cat. No. 100/123/515; Smiths Medical) as the intubation aids. These laryngoscopes and airway adjuncts were selected because these devices are used in the clinical setting at our study site. The endotracheal tube was lubricated with Airway Lubricant Spray (Cat. No. 252090; Laerdal Medical, Stavanger, Norway) before laryngoscopy, and a self-inflating bag (Cat. No. 87005340; Laerdal Medical) was placed within reach of the participant.

A high-fidelity airway management simulator (Difficult Airway Management Simulator Evaluation System, Cat. No. MW11 11,390–000; Kyoto Kagaku, Kyoto, Japan) was used to measure the forces applied on the maxillary incisors and tongue during ETI attempts. The implanted sensors in the simulator automatically quantified the forces applied on its incisor and tongue during laryngoscopy. This simulator can create a difficult airway by limiting mouth opening and cervical mobility. However, because novices are less likely to perform ETI in patients with trismus and cervical spine immobility at our ED, the “normal airway” mode of the simulator without restricted mouth opening and cervical mobility was chosen in this study. The time to ETI, defined as the time from the first contact with the device until the first successful lung ventilation, was also measured. The time to ETI was also automatically recorded by the implemented sensors in the simulator, which detect effective lung ventilation.

### Simulation scenario and study protocol

**Fig. 1:**
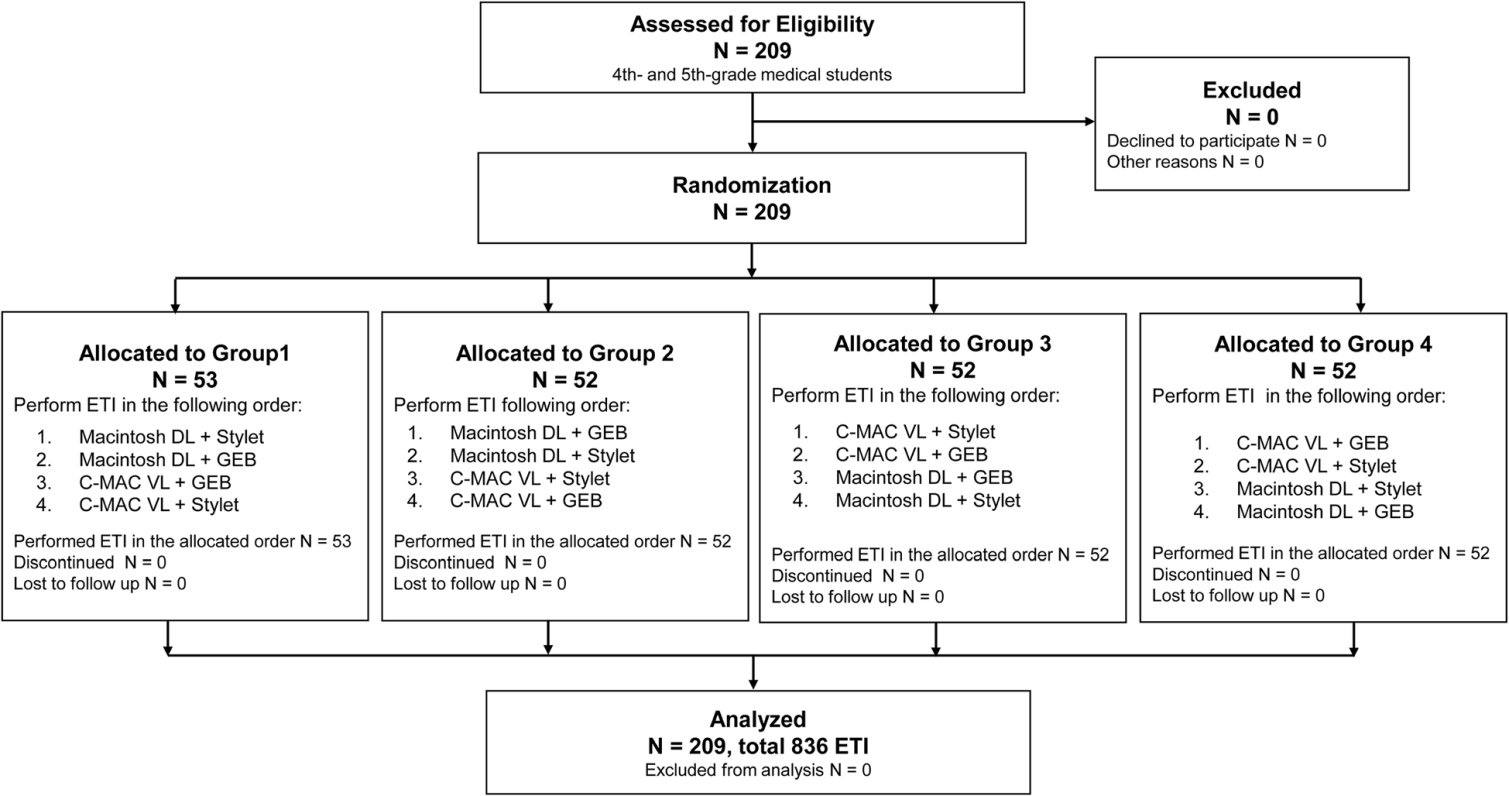
Participant flow diagram. DL: direct laryngoscope; ETI: endotracheal intubation; GEB: gum-elastic bougie; VL: video laryngoscope

First, all participants were given a standardized lecture and hands-on session using each laryngoscope and intubation aid by one of the investigators (Y.O.). This included oral instructions on ETI preparation and how to use each device as well as a demonstration of the intubation technique with each device. For acclimatization, each participant was then allowed to practice achieving one successful ETI with each laryngoscope and airway adjunct on the simulator. After this training session, each participant followed four simulation scenarios: they attempted ETI on the simulator using the (1) Macintosh DL and stylet, (2) Macintosh DL and GEB, (3) C-MAC VL and stylet, and (4) C-MAC VL and GEB. To minimize the learning curve effect, the order of the first ETI method was randomized for each participant by an online program (Research Randomizer, available at www.randomizer.org). With the aid of the program, the participants were divided into four groups as shown in Fig. 1. Our study had 2 levels of the primary factor (GEB and endotracheal tube + stylet) and 2 levels of the secondary factor (Macintosh DL and C-MAC VL), creating 4! (= 24) factor combinations. However, because it was very difficult to include 24 factor combinations, we decided to employ the balanced incomplete block design [20] with 4 trial arms. A recent simulation study also employed a similar randomization strategy [21].

In the endotracheal tube + stylet group, each participant attempted to intubate the trachea with an endotracheal tube + stylet with their preferred shape. In the GEB group, each participant attempted to pass the GEB into the tracheal inlet. If this was successful, an assistant loaded the endotracheal tube over the GEB, and the laryngoscopist guided the endotracheal tube through the vocal cords and into the trachea while keeping the laryngoscope in the mouth. After the trachea of the simulator was intubated, the cuff of the endotracheal tube was inflated with a 10-ml syringe (Cat. NO. SS-10ESZ20; Terumo, Tokyo, Japan), and each participant attempted to ventilate the lungs with a self-inflating bag. To reduce the effect of time zone differences on ETI [22], all measurements were conducted during a weekday morning.

### Outcome measures

The primary outcome measures were the maximum applied forces on the maxillary incisors and tongue during ETI attempts. The other outcomes of interest were the time to ETI, ETI success rate, and glottic view during intubation attempts. ETI success was defined as successful placement of the endotracheal tube into the trachea and confirmation of lung inflation within 120 s [16]. When the participants could not intubate the trachea within this period, the attempt was considered a failure and the ETI time was recorded as 120 s. The glottic view at each intubation attempt was scored by the participant using the Cormack–Lehane grading system [23]. Because the same researcher (Y.O.) was involved in the participant allocation, enrollment, and assignment processes as well as in the outcome measurements, the outcome assessment was not blinded.

### Power analysis

During the planning phase of this study, we attempted to estimate the potential improvement in the maximum applied forces on the oral structures during laryngoscopy with the test devices compared with the Macintosh DL. Previous studies estimated that the forces on the maxillary incisors and tongue with the Macintosh DL in a manikin ranged from 19.5 to 183 N and 6.3 to 40 N, respectively, depending on the experience of the laryngoscopist and the degree of difficulty [8, 16, 24–31]. The forces on the maxillary incisors and tongue can be changed by 0 to 100 N and 0 to 11 N, respectively, using various VLs and airway adjuncts [8, 16, 24–31]. These disparate data prevent precise sample size calculation in advance. Furthermore, to our knowledge, no previous study has employed medical students who are naive to ETI as study subjects to measure the forces applied during laryngoscopy. We therefore had to abandon our sample size estimation. Alternatively, we decided to use collectable data during the study period. The observed power after two-way analysis of variance was computed using the general linear model option of SPSS Statistics for Windows, version 22.0 (IBM Corp., Armonk, NY, USA). For this calculation, we referred to the relevant article [32] as well as the online contents from IBM Corp [33]..

### Statistical analysis

**Fig. 2:**
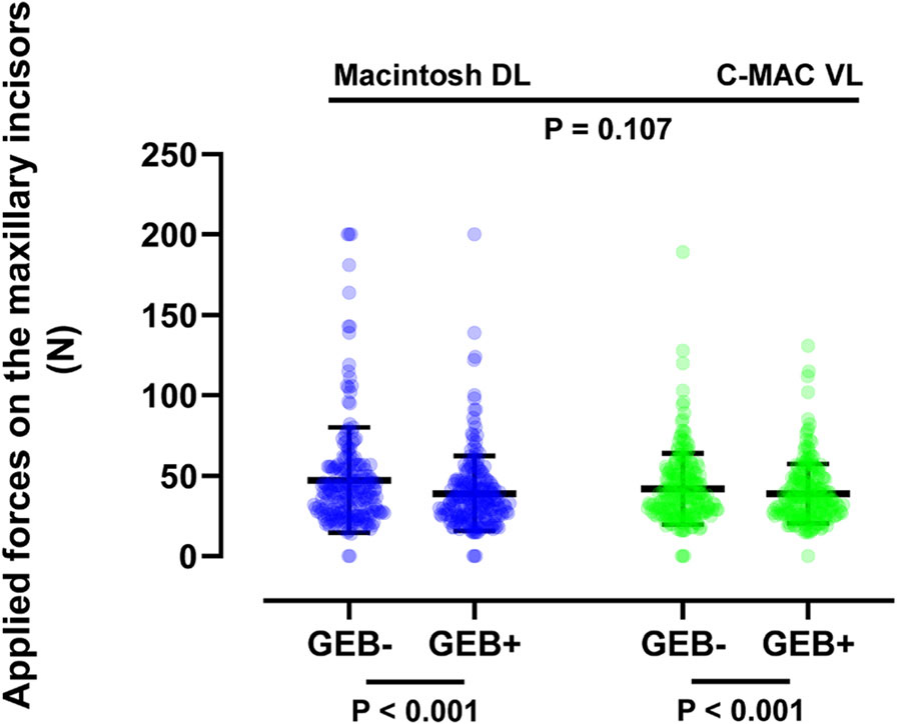
Comparison of maximum applied forces on maxillary incisors by each laryngoscope and airway adjunct. Column scatter plots representing the data distribution (circles), mean (horizontal bar), and standard deviation (vertical bar). The *P* values were derived from two-way analysis of variance. *n* = 209 in each group. DL: direct laryngoscope; ETI: endotracheal intubation; GEB: gum-elastic bougie; VL: video laryngoscope

**Fig. 3:**
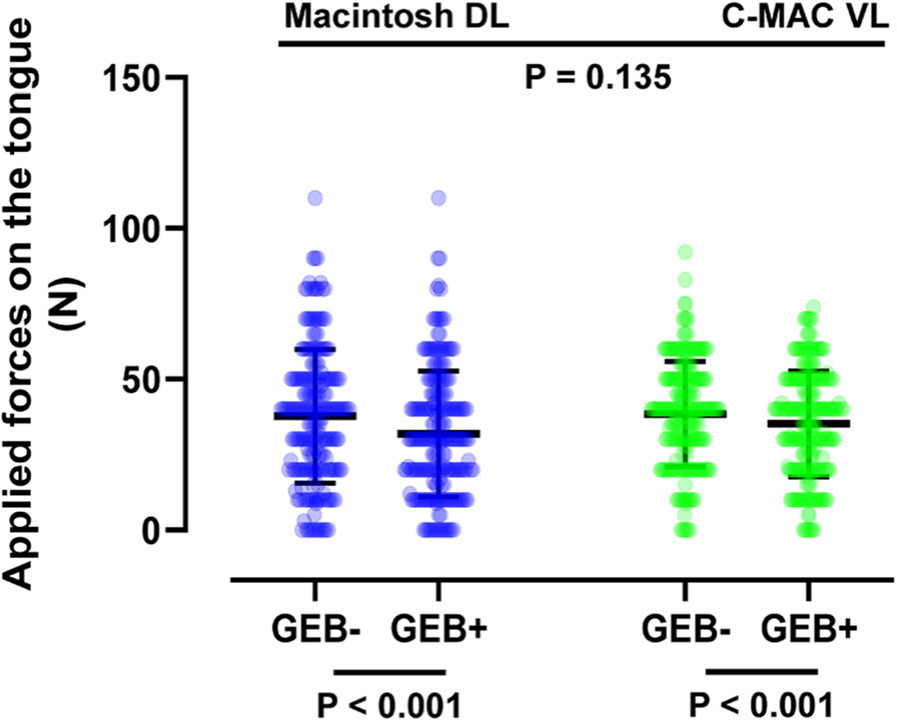
Comparison of maximum applied forces on tongue by each laryngoscope and airway adjunct. Column scatter plots representing the data distribution (circles), mean (horizontal bar), and standard deviation (vertical bar). The P values were derived from two-way analysis of variance. n = 209 in each group. DL: direct laryngoscope; ETI: endotracheal intubation; GEB: gum-elastic bougie; VL: video laryngoscope

**Fig. 4:**
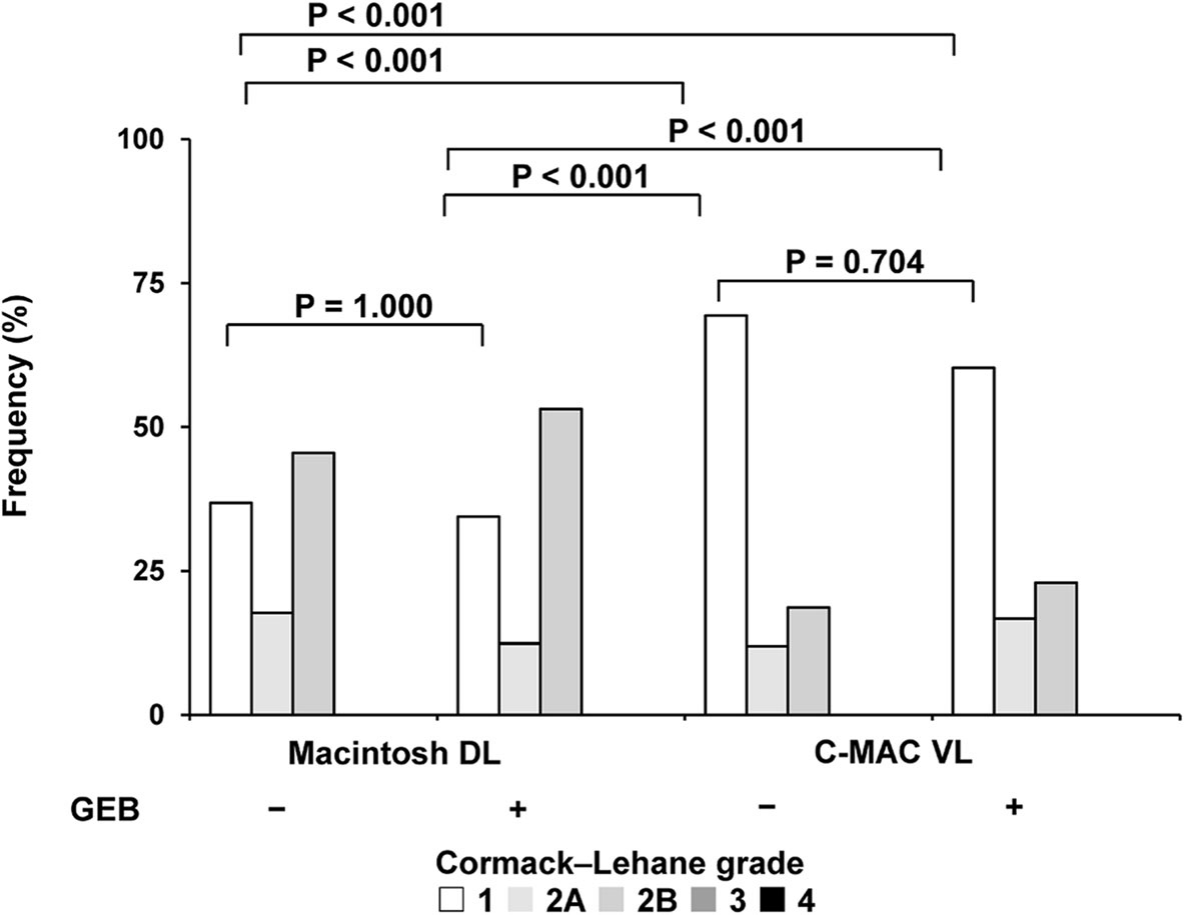
Distribution of Cormack–Lehane laryngoscopic view by each laryngoscope and airway adjunct. The P values were derived from the Kruskal–Wallis test with Bonferroni correction. n = 209 in each group. DL: direct laryngoscope; ETI: endotracheal intubation; GEB: gum-elastic bougie; VL: video laryngoscope

The statistical analysis plan was determined a priori. Our sample was relatively large (*N* = 209, total of 836 recorded ETI attempts) compared with previous studies [8, 16, 24–31], and only the “normal airway” mode was used; thus, we expected a normal distribution of continuous variables such as the maximum applied forces on the oral structures and the time to ETI. Two-way analysis of variance was therefore employed to look for a correlation between these outcomes and each device. Between-group differences in ordinal scales, such as the Cormack–Lehane grades, were compared using the Kruskal–Wallis test with Bonferroni correction. Between-group differences in categorical variables, such as the ETI success rate, were compared using the chi-squared test. All statistical analyses were performed using SPSS Statistics for Windows, version 22.0. The column scatter plots shown in Figs. 2, 3, and 4 were created using GraphPad Prism 8 (GraphPad Software, San Diego, CA, USA). A *P* value of < 0.05 was considered to indicate statistical significance.

## Results

### Study participants

From April 2017 through March 2019, 209 medical students without prior ETI experience were recruited in this study (Fig. 1). All participants received the intended standardized lecture and training session, performed ETI in the allocated order, and were included in the analysis of the primary outcomes. Their mean age was 23.7 ± 2.0 years, and 30.6% (64 of 209) were female.

### Primary outcomes

As shown in Fig. 2, the maximum force applied on the maxillary incisors was significantly lower when using a GEB than when using an endotracheal tube + stylet both with the Macintosh DL (39.0 ± 23.3 vs. 47.4 ± 32.6 N, *P* < 0.001) and C-MAC VL (38.9 ± 18.6 vs. 42.0 ± 22.1 N, P < 0.001). Similarly, as shown in Fig. 3, the force applied on the tongue was significantly lower when using a GEB than when using an endotracheal tube + stylet both with the Macintosh DL (31.9 ± 20.8 vs. 37.8 ± 22.2 N, *P* < 0.001) and C-MAC VL (35.2 ± 17.5 vs. 38.4 ± 17.5 N, *P* < 0.001). Use of the C-MAC VL did not significantly affect the forces applied on the maxillary incisors and tongue (Figs. 2 and 3).

### Other outcomes of interests

**Fig. 5:**
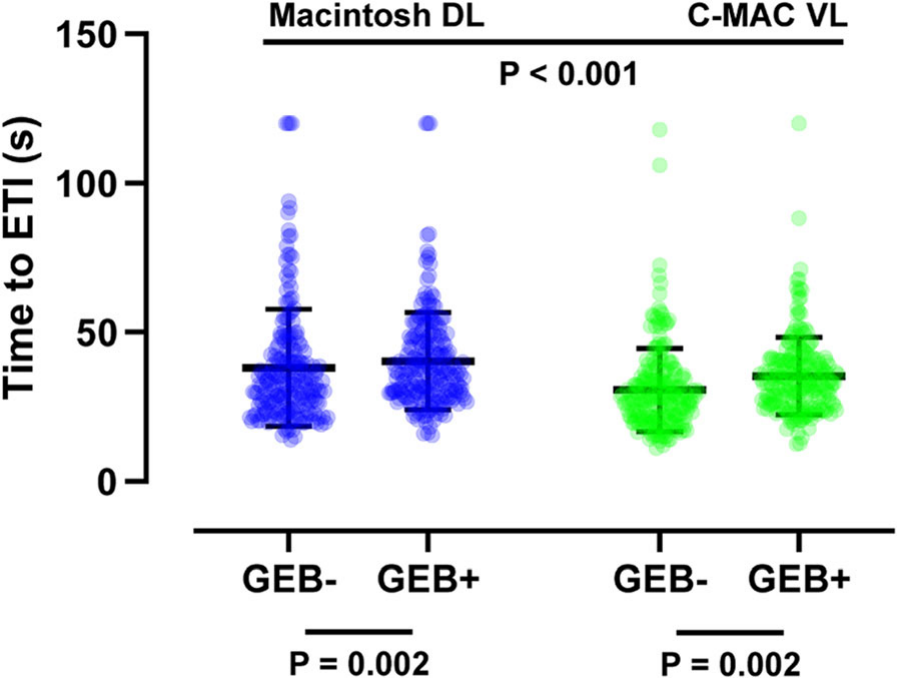
Comparison of time to ETI by each laryngoscope and airway adjunct. The time to ETI was defined as the time from the first contact with the device until the first successful lung ventilation. The column scatter plots represent the data distribution (circles), mean (horizontal bar), and standard deviation (vertical bar). The P values were derived from two-way analysis of variance. n = 209 in each group. DL: direct laryngoscope; ETI: endotracheal intubation; GEB: gum-elastic bougie; VL: video laryngoscope

As shown in Table 1, when using an endotracheal tube + stylet, the ETI success rate was significantly higher with the C-MAC VL than Macintosh DL (100% vs. 98.1%, *P* = 0.044); however, this significantly higher success rate was not observed when using a GEB (99.5% vs. 99.0%, *P* = 0.562). The use of a GEB did not affect the ETI success rate either with the Macintosh DL or C-MAC VL (Table 2). The C-MAC VL significantly improved the laryngoscopic view (all P < 0.001) (Fig. 4) and reduced the time to ETI (P < 0.001) (Fig. 5) compared with the Macintosh DL regardless of GEB use. In contrast, the use of a GEB did not improve the laryngoscopic view (Fig. 4) and was associated with a significantly extended time to ETI compared with the use of an endotracheal tube + stylet both with the Macintosh DL (40.5 ± 16.3 vs. 38.2 ± 19.6 s, *P* = 0.002) and C-MAC VL (35.5 ± 12.9 vs. 30.8 ± 14.0 s, P = 0.002) (Fig. 5).

**Table 1:** ETI success rate by each laryngoscope

	Macintosh DL, successful/total attempts	C-MAC VL, successful/total attempts	P*
Endotracheal tube + stylet	205/209 (98.1)	209/209 (100)	0.044
GEB	207/209 (99.0)	208/209 (99.5)	0.562

Data are presented as n (%).

ETI success was defined as successful placement of an endotracheal tube into the trachea with confirmation of lung inflation within 120 s.

*Chi-squared test

DL: direct laryngoscope; ETI: endotracheal intubation; GEB: gum-elastic bougie; VL: video laryngoscope

**Table 2:** ETI success rate by each airway adjunct

	Endotracheal tube + stylet, successful/total attempts	GEB, successful/total attempts	P*
Macintosh DL	205/209 (98.1)	207/209 (99.0)	0.411
C-MAC VL	209/209 (100)	208/209 (99.5)	0.371

Data are presented as n (%).

ETI success was defined as successful placement of an endotracheal tube into the trachea with confirmation of lung inflation within 120 s.

*Chi-squared test

DL: direct laryngoscope; ETI: endotracheal intubation; GEB: gum-elastic bougie; VL: video laryngoscope

## Discussion

This prospective crossover study showed that the use of a GEB reduced the force applied on the maxillary incisors and tongue as compared with an endotracheal tube + stylet during laryngoscopy both with the Macintosh DL and C-MAC VL among novices. However, the use of a GEB was also associated with a longer time to ETI than was the use of an endotracheal tube + stylet regardless of the type of laryngoscope. We also observed that the C-MAC VL significantly improved the glottic view and time to ETI on the high-fidelity simulator as compared with the Macintosh DL.

Applying excessive forces on the oral structures during laryngoscopy is associated with several ETI-related complications. For example, excess force on the maxillary incisors increases the risk of dental injury, which is the most common medico-legal claim against laryngoscopists [34, 35]. Excess force on the upper airway during laryngoscopy also increases sympathetic activities [36, 37], causing adverse hemodynamic alterations such as hypertension, tachycardia, arrhythmia, and even cardiac arrest [1–5]. Therefore, ETI should be performed with minimal force applied on the upper airway to prevent these consequences. In this study, compared with an endotracheal tube + stylet, the use of a GEB was associated with lower maximum forces on both the maxillary incisors and tongue during both direct and indirect laryngoscopy among novices. There are several plausible reasons for these observed findings. First, the GEB has a smaller diameter than an endotracheal tube. Thus, the GEB may obscure less of the laryngoscopist’s view of the glottic inlet as it approaches, allowing the trachea to be intubated with application of less force. A previous study showed that a GEB was especially useful when such an incomplete view of the glottis is obtained [12, 13]. In the present study, nearly two-thirds of direct laryngoscopy procedures and one-third of indirect laryngoscopy procedures resulted in a Cormack–Lehane grade 2A or 2B view. Similarly, the angled tip of a GEB may enter the limited glottic opening more easily than an endotracheal tube + stylet, with less lifting force. Our findings are consistent with a previous observation by Hung et al. [16], who found that the use of a GEB during ETI attempts was associated with less lifting force than the use of an endotracheal tube + stylet in a simulated difficult airway created by manual in-line stabilization among anesthesiologists. Based on these observations, we speculate that the GEB also reduces the forces applied on the oral structures in other types of difficult airways, such as trismus, micrognathias, tongue edema, and epiglottitis, even for experienced operators. The effect of a GEB on the force applied during laryngoscopy in such conditions warrants further investigation. A previous clinical study also showed that routine GEB use was associated with an increased first-attempt success rate among patients who underwent emergency ETI in the ED [12]. These results and our finding that a GEB has an incremental benefit in improving the force applied on the oral structures during ETI attempts collectively suggest the expanded role of a GEB beyond an airway adjunct for difficult airways and a rescue device after failed ETI attempts.

By employing medical students as participants in the present study, we found that the average maximum force applied on the maxillary incisors during ETI attempts using an endotracheal tube + stylet was 47.4 N with the Macintosh DL and 42.0 N with the C-MAC VL. These forces were higher than those shown in previous studies. For example, using the same simulator and resident physicians, Nakanishi et al. [24] and Takeuchi et al. [8] reported that the average maximum force applied on the maxillary incisors during ETI attempts was 19.5 to 28.0 N when using the Macintosh DL and 0.0 to 22.0 N when using the other type of VL. In a randomized clinical trial including 141 patients with non-anticipated difficult airways, Pieters et al. [31] found that the average maximum force applied on the maxillary incisors during ETI attempts among anesthesiologists was 30.2 N with the Macintosh DL and 1.2 to 16.2 N with various type of VLs. We consider that the discrepancies between our results and those of past studies were due to the difference in the participants’ clinical ETI experience and skill proficiency. These findings collectively suggest that the force applied on oral structures can be quantified as a marker of ETI skill proficiency. As previously noted, evaluation of the applied forces on oral structures during ETI attempts using a simulator may be helpful to monitor the ETI skill development among novices [8]. Importantly, the use of a simulation-based assessment system is harmless and repeatable.

Another previous study demonstrated that the complete arch of the incisors has a maximum bite force ranging from 150 to 200 N [38]. Therefore, if the teeth are healthy, a peak force on the maxillary incisors of more than 150 to 200 N might be a risk factor for dental injury during laryngoscopy. Nevertheless, if the patient has preexisting dental problems such as dental caries, periodontal disease, or abnormally positioned teeth, dental injury can be caused more easily [35] with much less lifting force. Therefore, the force applied during laryngoscopy in the ED should be minimized. Actually, “minimal” force applied on the oral structures to prevent ETI-related complications has not been well defined [8]. The present study showed that use of a GEB had the potential to reduce the force applied on the oral structures by approximately 20% during direct laryngoscopy and 10% during indirect laryngoscopy among novices. The clinical significance and relevance of these findings should be confirmed in future research.

In this study, the use of a GEB was also associated with an extended time to ETI as compared with an endotracheal tube + stylet regardless of the type of laryngoscope used. Because GEB-assisted ETI requires the additional step of placing the endotracheal tube over the GEB, the duration of ETI with a GEB may be longer than that with an endotracheal tube + stylet among medical students without prior experience of its use. In contrast, a recent randomized clinical study showed that among emergency physicians, the use of a GEB resulted in a significantly shorter ETI time than the use of an endotracheal tube + stylet in patients with a difficult airway [13]. Other studies showed that the use of a GEB by anesthesiologists also reduced the ETI time in the operation room setting [39], simulated difficult airway in infants [40], and simulated cardiopulmonary resuscitation [41]. These discrepancies between our results and past studies can likely be explained by differences in clinical experience and training levels. Senior physicians who supervise ETI should note the possibility that a GEB may increase the time to complete ETI if used by novices.

In line with previous studies [42–47], the C-MAC VL significantly improved the glottic view and time to ETI on the high-fidelity simulator as compared with the Macintosh DL. A past clinical study also showed that novice healthcare providers conduct ETI more successfully and safely with use of the C-MAC VL than Macintosh DL [44]. In a helicopter emergency medical system, the first-attempt ETI success rate was improved after introduction of a new intubation protocol combining the C-MAC VL and a GEB [45]. In addition, the video monitor of the C-MAC VL enables the supervising physician to more easily share the glottic view and confirm successful ETI. These results, together with our data, support the usefulness of the C-MAC VL with a GEB in novice laryngoscopists.

### Limitations and strengths

This study had five major limitations. First, we only measured the force applied on the oral structure using simulators. Like many previous studies [8, 16, 24–31], we were therefore unable to directly show the association between each intubation device and ETI-related complications. Nevertheless, ample evidence suggests that an association exists between excess force applied on the oral structures during laryngoscopy and the development of ETI-related adverse events [6–8, 35–37].

Second, we did not calculate the sample size in advance. As described in the Methods section, we were unable to estimate the precise sample size because of disparities with previous data [8, 16, 24–31]. However, the post hoc power calculation demonstrated that the power of our study was sufficient (power > 0.90) for all primary outcomes examined. In fact, our sample is largest among similar simulation studies. This study showed how use of the C-MAC VL and a GEB affected the maximum forces applied on the oral structures during laryngoscopy performed by a novice compared with the Macintosh DL. We believe that this information can be used in the effect size estimation of each intubation device, establishing a baseline for future clinical studies.

Third, our study was simulation-based; therefore, our findings may not necessarily reflect the outcomes in clinical settings. As previously noted [8, 16, 24–31, 48], various factors may interrupt ETI attempts in clinical practice (e.g., overwhelming stress in living patients, chest compressions, noisy and uncontrolled environment, secretions and bleeding, and blurred VL images caused by fogging). However, quantitative measurement of the force applied on the oral structures during ETI is difficult, especially when supervising novice physicians. Before direct clinical evaluations, we first performed validations with the simulator.

Fourth, because the participants were medical students, our findings may not be extrapolated to ETI attempts by experienced operators. We did not design this study to determine which laryngoscopes and airway adjuncts are most effective for skilled laryngoscopists because experts are likely to use their preferred intubation device.

Finally, because the same researcher was involved in participant allocation, enrollment, and assignment as well as outcome measurement, there is a theoretical risk of biased assessment. However, all primary and secondary outcomes except for the glottic view were automatically quantified by the implanted sensor in the simulator. This objective measurement system mitigates the concern of bias.

In spite of these limitations, this study also had several strengths. First, because the participants had homogenous backgrounds with no clinical ETI experience, and because this study was designed as a randomized crossover study, we believe that our results solely demonstrate the differences in intubation devices.

Second, to the best of our knowledge, our findings are the first to demonstrate the association between the force applied on the oral structures and the use of a GEB in ETI performed by novices. The GEB enables novices to perform ETI with less force applied on the oral structures; this may in turn reduce the complications associated with ETI compared with an endotracheal tube + stylet. Our results can be used to help senior physicians choose the most effective devices when supervising ETI performed by novices.

## Conclusions

In this prospective crossover study using a high-fidelity simulator, we found that compared with the use of an endotracheal tube + stylet, the use of a GEB was associated with lower maximum forces applied on the oral structures during both direct and indirect laryngoscopy performed by novices. We also found that use of the C-MAC VL was associated with an improved glottic view and reduced ETI time. Our results suggest the expanded role of a GEB beyond an airway adjunct for difficult airways and a rescue device after failed ETI attempts.

## Data Availability

All data relevant to the study are included in this published article. Further datasets analyzed during the study are available from the corresponding author on reasonable request.

## Abbreviations

DL: direct laryngoscope
ED: emergency department
ETI: endotracheal intubation
GEB: gum-elastic bougie
VL: video laryngoscope
